# Intra-Household Allocation of Nutrients in an Opening China

**DOI:** 10.3390/ijerph15040700

**Published:** 2018-04-09

**Authors:** Li Zhou, Xiaohong Chen, Lei Lei

**Affiliations:** 1 College of Economics and Management, Nanjing Agricultural University, Nanjing 210095, Jiangsu, China; 2017206014@njau.edu.cn; 2 Institute of Developing Economies, Japan External Trade Organization, Chiba 261-8545, Japan

**Keywords:** nutrition, intra-household, foreign direct investment, China

## Abstract

This paper uses China Health and Nutrition Survey (CHNS) data to analyze the effect of foreign direct investment (FDI) on nutrient intakes across various family roles to identify the different family roles’ heterogeneous nutrition intake responses to economic openness. The empirical evidence shows that FDI enhances labor forces’ calorie intake significantly, especially for rural households. The government should continue facilitating more FDI inflows, especially FDI in secondary industries for rural populations. However, the larger the family, the smaller the effect of FDI on nutrient intake for some family roles. The elderly and children may be weaker responders on nutrient intake than other family members in an open economy. This implies the existence of intra-household redistribution and that the level of effectiveness will decrease with family size. The results suggest that family members in rural areas can benefit more in terms of nutrient intake. Our empirical evidence also indicates that female family members’ calorie intake from the FDI effect is higher than that of male family members (except for the granddaughter/grandson). Preferential policies should be provided for the FDI, flowing to rural areas and female dominant industries.

## 1. Introduction

Since its reform and opening in 1978, China has experienced improvements in food consumption and nutrition intake. According to the China Nutrition and Chronic Diseases Status Report (2015) (Abbreviated CNCDSR in the following text), the average calorie intake of the Chinese population in 2012 was 2172 kcal (consisting of 301 g of carbohydrate, 80 g of fat, and 65 g of protein). Nutrient intake is vital for health promotion, social equity, and long-term economic development. Direct economic losses caused by malnutrition are estimated to be in the range of 3 to 5% of GDP in developing countries [1]. Therefore, it is imperative to conduct research on nutrition intake and transition.

Debate regarding the relationship between calorie and income has been widely discussed in various studies [2,3]. Bouis and Haddad [4] measured how household calorie intakes have changed with income and estimated elasticities, which range from 0.08 to 0.14. Tian and Yu [3] reported that calorie intake increases with income growth, but with decreasing marginal returns. Aromolaran [5] revealed that increasing women’s household income share decreases household per capita calorie intake in low-income households in rural southwestern Nigeria using instrumental variable (IV) methods. This finding implies that food calorie intake responds negatively to a reallocation of household income from men to women.

Some studies have documented the impact of economic openness (globalization) on nutrition transition. Rayner et al. [6] found that trade liberalization can affect the food supply chain via factors such as food imports, exports, and FDI in food processing. They used FDI, supermarketization, and cultural change to illustrate complex linkages between trade liberalization and diet transition. Thow [7] studied the complex relationship between trade policy and nutrition transition. On the one hand, by increasing the availability and affordability of processed food and animal products, trade liberalization policies could facilitate nutrition transition in developing countries [7,8]. On the other hand, the dietary patterns resulting from nutrition transition are associated with diet-related chronic diseases [7]. This means that, although globalization has the potential to improve nutrition, some aspects of the globalization process may deteriorate human nutrition and health [9]. Globalization has played an important role in changing energy consumption patterns, dietary intake, and resulting diseases in the world [10]. However, most of these studies lack empirical analysis based on microdata. A number of related studies have explored the impact of economic openness on health with mixed findings. For example, Vogli et al. [11] and Burns et al. [12] found that FDI is positively associated with BMI and other health indices. However, Kawachi [13] identified a negative relationship between economic globalization and labor health.

Moreover, the impact of economic openness on different individuals’ nutrition intake is often neglected. In particular, there is no study focusing on intrahousehold individual nutrition intake from the impact of economic openness perspective, as far as we know. Mussa [14] found that intrahousehold nutrition inequalities are more pervasive than interhousehold ones. The evidence of intrahousehold bargaining power may explain the inequality to some extent [15,16]. There is a reallocation effect between family members within each household [17,18,19,20]. Typical examples include situations in which surplus laborers in rural households migrate to seek more off-farm income [21] and regularly send their salaries home to support their families, which affects their nutrition intake, and mothers’ economic independence possibly benefiting their children [18]. The intrahousehold reallocation effect may protect vulnerable people, such as the elderly, children, unhealthy, and disabled people. Parents distribute household resources to their children because of altruism [22,23]. Baeten et al. [24] noted that seniors over 70 years old were still supported by family-based self-insurance mechanisms in rural China. However, different family roles may have different outcomes in nutrition reallocation. For example, Shimokawa [23] found a strong gender bias against girls in cities while children and the elderly were both affected in rural areas when allocating intrahousehold calories. In previous studies, researchers analyzed this intrahousehold reallocation with a negative shock. For example, Carson et al. [25] explained that intrahousehold reallocation of working hours between family members are used to reduce the potential risk induced by arsenic exposure. In contrast, our paper will test the effect with a positive shock (i.e., the impact of economic openness on intrahousehold nutrient intake).

We will utilize the CHNS data to analyze the effect of FDI on nutrient intake across various family roles to identify the different family roles’ heterogeneous nutrition intake responses to economic openness. Following previous studies, this paper will use key factors such as gender, marital status, family responsibilities, social connections, and social status [26,27] to study this topic. With this thorough analysis, more targeted food policies and economic development policies can be formulated. The paper proceeds as follows. The next section introduces empirical models and variables. Section 3 presents data and descriptive statistics of the sample. Section 4 provides econometric results with discussion, and the final section draws conclusions and policy implications.

## 2. Methods and Variables

It is believed that economic openness changes nutrition intake through both income and non-income effects. For the nutrient intake of the income effect, Braunstein and Brenner [28] found that FDI could have a positive impact on individual income. Researchers have shown that the impact of economic openness on income varies across genders and regions. For example, Chen et al. [29] noted that globalization could encourage female employment and reduce gender discrimination, which is beneficial for female income improvement. Rising earnings resulting from FDI openness may be beneficial to food consumption and nutrition. The non-income effect on nutrient intake mainly refers to the change in eating habits. Influenced by the Western diet with more fat, more animal products, and high-energy density foods, the Chinese traditional diet, with a focus on grain and plant products, has been changing [30]. Urbanization and globalization may increase consumption of non-traditional foods, such as processed foods [10,31]. Dietary patterns are affected by price changes, production practices, and the presence of trade and markets in the developing world [31]. FDI may also make more highly processed foods available to more people by lowering prices, establishing new purchasing channels, optimizing the effectiveness of marketing and advertising, and increasing sales [9]. Therefore, we speculate that FDI-nutrient intake coefficients are significantly positive both in urban and rural areas.

*Hypothesis* *1*: DI openness would increase intrahousehold nutrient intake in both urban and rural areas.

Those with labor force roles could obtain higher income levels than other members, which means a higher bargaining power in the household. The intrahousehold reallocation effect on food consumption and nutrient intake may protect non-labor force members who are more vulnerable (e.g., children and the elderly). Therefore, labor force roles may consume more nutrients for both higher intrahousehold bargaining power and higher labor supply intensity under the impact of FDI openness. Another Hypothesis 2 is assumed as follows.

*Hypothesis* *2*: The positive nutrient intake coefficients of labor force roles are higher than other family roles in an open economy.

To study the regional effect, we will adopt subsample regression disaggregated by rural and urban areas. Roemling and Qaim [32] noted that food choices, job types, and personal hobbies in urban areas are quite different from those in rural areas. Burggraf et al. [33] separated rural and urban samples when analyzing nutrition transition in China and found considerable elasticity differences between them. This paper will not only split the sample by region, into urban and rural, but will further split the sample by family role into the husband sample, wife sample, son sample, and so on. The purpose of splitting samples by family role is to capture the different nutrition intake changes of each family role responding to economic openness.

(1) The family role regression

This study focuses on ten family roles based on the traditional Chinese family structure: husband, wife, father, mother, son, daughter, son-in-law, daughter-in-law, grandson, and granddaughter. Each family role’s sample regression is based on the equation below:

(1) $$N_{i}=\alpha_{i}+\beta_{1}FDI+\beta_{2}Z_{i}+\beta_{3}Dummy+\varepsilon_{i}$$

In Equation (1), subscript *i* refers to a specific family role group. The term $\alpha_{i}$ is a time-invariant and group-specific unobserved term, and $\varepsilon_{i}$ is a random error term. Variable *N* is the three-day average calorie intake of each family role group. For measuring nutrition, calorie intake quantity changes have been widely utilized [34,35]. Hence, calorie intake (calorie) is adopted as the core dependent variable here because it measures the energy provided from all nutrients and contains more information about the nutritional status [36].

**Figure 1:**
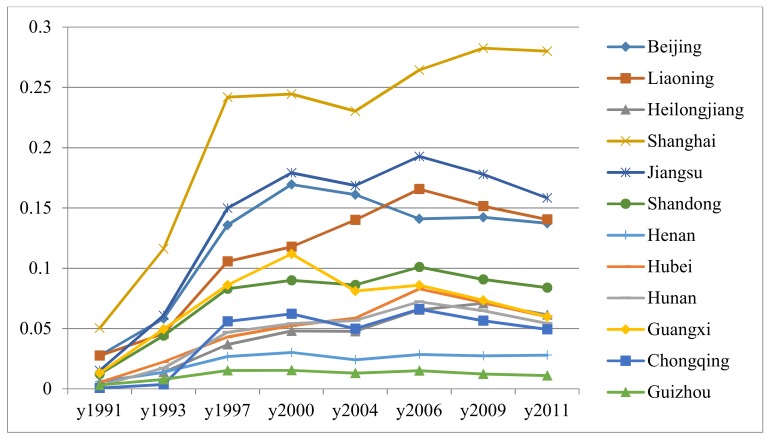
FDI openness statistics across sample regions and years.

FDI openness is used as a proxy of economic openness. The accumulated FDI stock divided by GDP of each province and each sample year measures FDI openness. The variable has been converted by the official exchange rate, accumulated consumption price index (CPI) based on the year 2011, and annual depreciation rate (9.6%). The FDI stock variables and initial values are constructed following previous studies [37,38]. Exchange rate conversion is necessary since the unit of FDI is dollars while GDP is measured in CNY. The accumulated consumption price index (CPI) could eliminate price factors since FDI contains price fluctuations. According to the estimation of Zhang et al. [39] of China’s provincial capital stock depreciation, FDI stock needs to get rid of 9.6% depreciation. Figure 1 provides the trends in *FDI* variable statistics across sample regions and years.

Age, education, and BMI are used to capture individual demographic characteristics [14,32,40,41]. In addition, gender and education level are the main variables that reflect the bargaining power of each family role [15,32]. Therefore, variables controlling individual and family characteristic include age (*Age*), education (*Edu*) level, body mass index (*BMI*), family size (*Fsize*), and total household income (*Hinc*). The definition of family roles in our model automatically implies the gender characteristics, so, to avoid redundancy, it is not included here. The family size variable should be controlled since it measures intrahousehold resource availability and allocation issues about nutrition [41]. We speculate that the family size variable could affect intrahousehold food consumption and nutrient intake.

The regional price factor involves a series of accumulated food price indices (*Pgrain*, *Poil*, *Pmeat*, *Pegg*, *Paquatic,* and *Pvegetable*). Food price reduction increases poor people’s access to food [33] and has a positive impact on people’s nutritional well-being [42]. Bhargava [43] also documented that high food price led to an energy intake reduction. Thus, this study calculated the accumulated food price indices of grains, oil, meat, eggs, aquatic products, and vegetables to capture the price effect on nutrition. The accumulated food price is calculated by consumption price indices of each province in the base year 2011. To show the calculation process, this study takes the 1991 Beijing grain price index as an example. With the price index of 2011 set to 1, we first multiply the Beijing grain CPI across 1991 to 2010. Then, dividing the 1991 Beijing grain CPI by the multiplied results, we obtain the converted Beijing 1991 grain CPI at the base year 2011. The province-specific effect and time-fixed effect are controlled using dummies of twelve regions and eight waves of survey data in the sample period from 1991 to 2011 in China.

(2) The joint consumption regression

Intrahousehold decisions on food may affect each family member’s calorie intake [44]. To examine the responses of intrafamily members’ nutrient intake under the impact of FDI, it is critical to consider the intrahousehold joint nutrient consumption decision. Within a household, people are related by blood and kinship. They live together, pool all or most of their income for living, and generally share the same food supply [22]. Therefore, intrahousehold food is the main nutrition source for each individual member of a family. The FDI is used as the external shock in this paper to study nutrient intake responses. When we control the household total income, the response of each family member’s nutrient intake to FDI might be affected by two factors: (a) the family utility maximization function [20] and (b) family members’ bargaining power reflected by factors such as individual income or education level [16]. Senauer and Jacinto [22] introduced two household economic models to incorporate both factors. One model is to maximize the household joint utility function under the budget constraint. The other is based on the bargaining effect to reconcile the differences across family members. In our model, the household income is used as the budget constraint condition. Education level and individual income are used to reflect bargaining power.

A multivariate regression model is employed to explore the nutrient intake variations of subgroups since residuals of each family member are correlated. The model is the same as the above equation, but the joint consumption decision is considered, instead of the individual’s decision [45]. For each family member, the left side of the equation is the nutrient intake of all members of the family. Their personal characteristics (age, education level, and BMI index) are added to the control vector (*Z*) on the right side of the equation. The remaining variables remain the same as in the family role’s regression. Correlation coefficients of residuals are also reported to capture the interrelation of different family members’ nutrient intake decisions. To further provide more detailed nutrient intake responses, we also use the consumption amount of protein (*Protein*), fat (*Fat*), and carbohydrate (*Carbo*), instead of the calorie intake amount, in the regression equation as independent variables. The amount of calorie, fat, protein, and carbohydrates provided by the CHNS dataset is calculated based on the Chinese Food Composition Table [46].

(3) The two-stage least squares (2SLS) regression for individual income effect

Here this study will analyze the individual income transmission effect of FDI on nutrient intake. FDI openness stimulates individuals’ earnings [28], which are directly linked to their food consumption and nutrient intake. The intrahousehold joint consumption effect was another factor in analyzing the income effect, since one family member’s nutrient intake may be indirectly affected by another’s. A typical example is the couple’s interaction. For example, the remittances between husband and wife may generate an invisible income source [47] that impacts the nutrient intake of both.

This study only focuses on prime-age adults who are between 18 and 60 years old when estimating the income effect. Based on the efficiency wage theory [48], we use instrument variables (IVs) for individual income to perform regressions by 2SLS. For IVs of income, we use each household’s asset value, adding the wage rate for different local jobs of the working family members in the household. The household assets here include agriculture appliances (*Ktrans*), professional appliances (*Kagr*), and transportation carriers (*Kprof*) in detail. The wage rate here includes the average daily wage of a male factory worker (*Wage_male*), female factory worker (*Wage_female*), domestic helper (*Wage_helper*), construction worker (*Wage_const*), and driver (*Wage_driver*) monthly income in the 2SLS analysis. This wage information of the most common job types in China is obtained from the CHNS community level survey. The variables *Ktrans*, *Kagr*, and *Kprof* are obtained at the household level, and the rest are at the community level. Those wage rates that relate to individual income depend closely on regional economic prosperity and are less relevant to personal nutrient intake decisions. The household business asset is adopted by Aromolaran [5] to instrument women’s income when studying its impact on women’s nutrition intake. You et al. [49] used the five-year average provincial annual growth rate of average wage (per worker) to deal with the endogeneity problem between income and nutrient intake. They illustrated that there is a strong link between personal wages and the provincial wage growth rate, and the provincial wage growth rate may not be related to personal nutrient intake decisions.

All variables are used in the logarithm form to level out the skewed distribution, except for dummies and food price indices, which are normalized. Table 1 provides the detailed definitions of each variable.

## 3. Data and Descriptive Statistics

### 3.1. Data

The paper uses panel data from eight survey waves (1991, 1993, 1997, 2000, 2004, 2006, 2009, and 2011) covering 20 years from the CHNS dataset. The CHNS is one of the most representative databases that includes intrahousehold individuals’ demographic and social features, as well as detailed nutrient intake information. The paper seeks to draw the representative intrahousehold sample that was obtained in a multi-phase, random cluster process from nine well-bedded provinces (Liaoning, Heilongjiang, Jiangsu, Shandong, Henan, Hubei, Hunan, Guangxi, and Guizhou) and three municipalities under the central government (Beijing, Shanghai, and Chongqing). The sample of 12 regions covers from the north to the south with various geographical features, GDP levels, health indicators, and dietary styles. It records all food that each household member consumed in detail, including food consumed away from home for three consecutive days on a 24-h recall basis. These records are transferred into nutrition values based on the Chinese Food Composition Table [46]. There are limitations of recall data due to potential inaccuracy in food consumption, including, for example, recall bias. The accuracy of data may also depend on the skills and probing abilities of the enumeration team. Besides, the design defect of the Food Composition Table may bias the calculation shares of carbohydrate, fat, and protein. These values cannot be totally precise, which may affect the result to some degree but can be controlled by our estimation methods. The household survey provides information on the individual income and labor time allocation of each family member. IVs for income are also obtained from the CHNS survey on the adult and on communities. Additionally, China Statistical Yearbooks provide food price indices of grains, oil, meat, eggs, aquatic product vegetables, GDP, and foreign direct investment data for the corresponding survey years.

There are more than 95,000 individual observations in the sample. Since CHNS does not provide detailed family role information, losing observations when we match up family members to get subsamples based on different family roles is unavoidable. For example, it is only possible to identify the observations of the oldest son, oldest daughter, oldest daughter-in-law, and oldest son-in-law, while their siblings’ information is missing in a household in the sample. Because there is no spouse information in the CHNS dataset, we regard the oldest son and oldest daughter-in-law as the couple in a household. Despite some errors, we assume that the matching between the son and daughter-in-law is sufficiently accurate in the intrahousehold joint regressions. There are four types of family compositions that are most common and representative in Chinese households: (a) two-member group with husband and wife (25,615 observations); (b) three-member groups comprising husband and wife with a son or daughter (10,296 and 7146 observations respectively); (c) four-member group, one comprised of husband, wife, son, and daughter (3190 observations) and the other comprised of husband, wife, son, and daughter-in-law (the daughter will move to her husband’s household if she gets married, so it is rare to see a son-in-law in a four-member group in China, 1975 observations); and (d) five-members groups, adding a grandson (486 observations) or a granddaughter (440 observations) to the second type of four-member groups (those groups account for 52.1%, 20.9%, 14.5%, 6.5%, 4.1%, 1%, and 0.9% of our total sample, respectively).

This study does not consider extended family (e.g., brother, sister, father-in-law, and mother-in-law) since the observations with an extended family are quite few. Throughout the research, husband refers to the male householder or female householder’s spouse, and wife refers to the female householder or male householder’s spouse. When we talk about son, daughter, and daughter-in-law, they are the oldest ones in the family, as mentioned earlier. The “oldest” is omitted for simplification.

### 3.2. Descriptive Statistics

Table 2 presents the summary statistics of all variables, using the pooled sample households of eight waves of data from 1991 to 2011. As Table 2 shows, total household income in our sample is about 32,945.51 CNY in urban areas and 22,730.82 CNY in rural areas. These are measured at the 2011 price level. The average household size is 4.01 in our sample, and total labor time averages 1530.68 h per year. Individuals consume 2189.16 kcal calories, 66.37 g proteins, 67.77 g fats, and 324.16 g carbohydrates per day on average in our sample. According to CNCDSR (2015), the average daily calorie consumption of an adult is 2172 kcal calories (55 g proteins, 80 g fats, and 301 g carbohydrates) in 2012. Accordingly, our sample average across 1991–2011 is close to the official records for 2012. Observations in the sample have 6.66 years of education on average, with the urban area having 1.79 more years than the rural area. The average BMI index in our sample is about 21.76, which is close to the average BMI index of 23.65 from CNCDSR (2015).

## 4. Results

### 4.1. Family Role Regressions

Table 3 shows regression results for the equation, which identifies the impact of *FDI* on calorie intake across various family members. From these tables, it is clear that economic openness through FDI has a positive and statistically significant impact on calorie intake at least at the 1% level for a husband, wife, and daughter-in-law, both in urban and rural areas. A 1% increase in FDI openness is followed by 0.084% and 0.096% increases in the calorie intake of the husband in urban and rural areas, respectively. For every 1% increase in FDI openness, the calorie intake of an urban and rural wife will increase 0.107% and 0.113%, respectively. For a daughter-in-law in the urban and rural areas, the corresponding increases are 0.147% and 0.111%, respectively. Additionally, a son and daughter in a rural area show a significant calorie intake improvement effect of FDI openness. More FDI inflows are accompanied by new technology and more skilled labor demand [50]. Adult members with higher education comprise the main labor supply in a family. They usually bear the economic burden of the whole family and thus may consume more calories than the children and elderly members in the household under the impact of FDI openness.

For each 1% increase in FDI, the calorie intakes of wife and daughter-in-law increase by 0.107% and 0.147% in urban areas, respectively, while the calorie intakes of husband and son increase by 0.084%, with no significant impact for a son. The FDI induced calorie intake increases of wife and daughter-in-law in a household are greater than that of husband and son in urban areas. Similarly, the calorie intake coefficients of the FDI effect for wife and daughter-in-law in rural areas are 0.113% and 0.111%, respectively, while the calorie intake coefficients of FDI effect for husband and son in the same area are 0.096% and 0.084%, respectively. This implies that females’ calorie intake in the family has improved more than their male counterparts with increasing FDI inflows. This result contradicts the previous findings of girls’ nutritional inequality versus boys in China [40] that girls have an adverse nutrient intake particularly in multiple children households.

The husband, wife, son, daughter, daughter-in-law, and son-in-law are the main prime-age adult members in the household. The elders (household head’s father and mother) and children (grandson and granddaughter) in a household have a relatively small and insignificant FDI effect on calorie intake. Gittelsohn [51] also found that the nutrient intake of the male and female children in rural Nepal remained the same under a variety of mechanisms. Those mechanisms include serving order, serving method, refusing to serve foods, channeling foods, and replacing low-status foods with high-status ones (for example, rice is regarded as superior to the other grains among the main staples (wheat, rice, and corn).

Additionally, in terms of household income-calorie elasticities, male members have higher elasticities than female members (not listed here). Mangyo [20] concluded that the elasticity of women’s nutrition intake is lower than that of men, and the elasticity of nutrition intake of elderly people is lower than that of other groups. These income-calorie elasticity values are negligible (close to zero) for both males and females, which is consistent with Behrman and Deolalikar’s [52] finding.

### 4.2. Joint Consumption Regressions

Table 4 and Table 5 provide the main regression results of the joint consumption decision for urban/rural sub-samples of different family structures, as discussed in Section 2 (the four types include two-member, three-member, four-member, and five-member groups):

(1) Based on joint consumption effect analysis, we find that FDI openness has a positive effect on the calorie intakes of husband, wife, son, daughter-in-law, and daughter with a significant sign, especially in rural areas. These findings are similar to the above results.

(2) In urban areas, FDI openness could improve carbohydrate and protein intake significantly while there is insignificant fat intake for some family roles. As family size increases, the nutrient intake of the FDI effect becomes insignificant. Kaushal and Muchomba [53] also reported that an increase in earnings resulting from food subsidies has a positive impact on calorie and protein intake but does not affect fat intake in the OLS models.

The results of three-member, four-member, and five-member groups in Table 4 indicate that sons and daughters have negative FDI effects on fat intake with significant signs. Other family roles show insignificant fat intake coefficients. The small sample sizes may be responsible for the insignificant results in four-member and five-member groups. Additionally, there are two possible reasons that account for the insignificant, even negative, fat intake coefficients in urban areas.

The income effect (health concerns) and labor intensity may be at work here. Tian and Yu [3] found that the share of calories from fat follows an inverse U-shape with income. This indicates that people tend to consume more healthy food with lower fat when their income is high enough, above a certain turning point. In our sample, the average income in urban areas is 1.53 times higher than that in rural areas. Urban younger adult groups, such as sons and daughters, who are mainly employed in knowledge-intensive industries, often have relatively higher incomes. The awareness of health problems resulting from the excess intake of fat, saturated fat, and cholesterol has a significant influence on individual nutrient intake [54]. With an increase in income, the health awareness of the urban young has become stronger than that of young people in rural areas. Health concerns could change the food consumption pattern by, for example, replacing animal fats and cholesterol with vegetable fats [10]. Therefore, the pursuit of health makes young people in urban areas consume a healthy diet with less oil and less fat, and even have a preference for a vegetarian diet in some cases. Those with young roles coming from rural areas are more likely to work in labor-intensive industries (such as agriculture). According to our calculation based on the sample year 2011, the highest proportions of occupation type are 19.83% “service worker” in urban areas and 47.11% “farmer, fisher, hunter” in rural areas. Those with higher labor intensity, such as farmer, may imply a comparatively stronger demand of rural people for meat consumption and fat intake. These reasons could explain why sons and daughters respond more significantly on fat intake than other members in urban families.

(3) For adults in a rural household, the coefficients of carbohydrate, fat, and protein response to FDI openness are positive and significant. Like the calorie analysis, FDI openness does not have a significant effect on the grandchildren’s carbohydrate, fat, or protein intake. Children, who are dependent on their parents and lack human capital, do not have an effective or timely response to FDI openness. Possible explanations for the insignificant nutrient intake results for the children roles (grandson and granddaughter) are as follows. According to our calculations of BMI index statistics (not shown), a lower BMI index value and higher share percentage of the children roles groups whose BMI indices are lower than BMI standards are found.

This phenomenon is more prominent, especially in rural China. A total of 75.14% of urban household heads and 86.28% of rural household heads have the family role of husband. Members who are far away from the household head fail to meet the Chinese BMI standards. The insignificant nutrient intake coefficients for grandchildren may be caused by their non-qualified BMI indices (unhealthy). It is the intrahousehold redistribution effect that supports those vulnerable roles (grandson and granddaughter) in food consumption and nutrient intake. In addition, the correlation coefficients of the residuals are stable between the couples (husband and wife, son, and daughter-in-law) in a four- or more member family at about 0.7 in all subsamples. This indicates that their decisions of calorie intake are significantly and positively correlated. The husband’s correlation coefficients with the next generation (son, daughter, and daughter-in-law) in a household are a little lower than the wife’s, which highlights the important role of the wife in a household. The other correlation coefficients are decreased successively by adjoining generations, but they are all above 0.4. These high and positive correlations reflect that the joint calorie consumption decision among different family members does matter to some degree.

### 4.3. Transmission Mechanism of Individual Income Effect

Table 6 and Table 7 show two-stage regression results with instrument variables. First, FDI could bring a positive impact on individual income [28] for both husband and wife in rural areas. However, the impact of FDI on individual income for urban family members is not statistically significant. Cheap labor forces from rural areas are employed more by foreign manufacturing industries to save cost. According to the investigation report of migrant labor released by the China National Bureau of Statistics in 2011, manufacturing was the major industry that migrant workers worked in that year, accounting for 36%. The China Statistics Yearbook showed that the proportion of FDI in the manufacturing sector was declining, but it remained as high as 45% in 2011. Therefore, given that most of the rural labor force is from the manufacturing sector, FDI could stimulate the growth of rural residents’ non-agriculture income. The proportion of rural residents’ income from non-agricultural income is growing. Raising the level of income and employment among rural families also increases the food quantities they can afford [8,55].

In addition, Taylor and Driffield [50] showed that FDI promotes the wage gap between skilled and unskilled labor in the UK manufacturing sector. Therefore, senior couples (husband and wife) can be more experienced and skilled with higher wages [56] than younger couples.

In the second step, FDI openness generates significant coefficients on calorie intake for a husband, wife, son, and daughter-in-law by using individual income rather than controlling household income as mentioned above. More family roles have a more significant nutrient intake coefficient in rural groups than urban groups, similar to the results in the above table. It is noteworthy that rural wives have a larger calorie intake coefficient of the FDI effect than their husbands with individual income in all groups.

Individual income also produces a positive and significant impact on one’s own calorie intake, especially as a wife, husband, and son in rural areas. Behrman and Deolalikar [57] used permanent income in their analysis and concluded with small income-nutrient elasticities as well. A 1% increase in a wife’s income leads to calories rising significantly for every member except the rural four-member I group. Gibson and Rozelle [58] also showed positive income-calorie elasticities. However, Senauer and Jacinto [22] reported that a wife’s wage rate could increase her own calorie intake but decrease her husband’s since more bargaining power is attached to the wife with a higher estimated wage. The result suggests that women’s independent income may benefit their children, which is supported by Dasgupta [18]. Bruce [59] also mentioned that women allocated more resources to maintaining health and improving the health of their dependents. More interestingly, a 1% increase in a husband’s income leads to 0.012%, 0.013%, and 0.016% calorie reductions significantly for the wife, son, and daughter-in-law in the rural four-member II group. The husband may not spend most of the income on food expenditures and nutrition improvement, while the wife may spend more on the household food supply, which increases every member’s calorie intake.

The income of husband and wife significantly reduces their children’s (son and daughter-in-law) calorie intake in four-members rural families but, for urban roles, increases their children’s intake. The absolute values of coefficients for husbands are stronger than for wives, indicating that fathers’ income affects children’s nutrient intake more [15].

### 4.4. Robustness Checks

#### 4.4.1. Regressions with Age Dummies

People in different age stages may have different nutrient intake responses to FDI openness. The nutrient intake status of adults and children may be affected in different ways due to dietary and lifestyle changes and limited nutrition and health knowledge [32]. According to the age division suggested by Chinese Dietary Guidelines [60], the continuous *age* variable is replaced with five age group dummies in the equation for a robustness check. The five age group dummies are preschool (2–5 years old), school age (6–17 years old), prime-age adult group (18–65 years old), elderly (66–80 years old), and senile old people group (above 80 years old).

The empirical results of the model using age dummies instead of the continuous age variable are reported in Appendix A Table A1. These results are similar to those of Table 4, indicating that the nutrient intake of the FDI effect remains significant for different age stages of each family role. Compared to their counterparts in the urban family, husband, wife, and daughter in the rural family consume more significantly for every 1% increase in FDI openness. Chen [61] noted that FDI helps to reduce urban-rural income inequality through employment creation and knowledge spillovers. It also contributes to economic growth. This implies that rural average incomes are enhanced more than those of urban areas under the impact of FDI openness. The nutrient intake coefficients of grandson and granddaughter still show no significant FDI effect. Roemling and Qaim [32] also found that children are more likely to be underweight than adults in dual burden households. The dual burden household means that, within the same household, children are underweight while adults members are overweight.

#### 4.4.2. Labor Time Controlled Effect

Labor time may influence an individual’s nutrient intake through physical activities. It is not necessary that all labor be physical work. Mental work would not involve too many physical activities. Therefore, an individual’s total labor time is controlled here in the multivariate regression model. Table A2 shows how FDI openness alters the calorie intake of each adult family on the joint consumption decisions after controlling the individual’s labor time. Due to insufficient residual degrees of freedom, results are not generated for the two five-member groups. Similarly, the calorie intakes of husband, wife, son, and daughter-in-law increase as FDI inflow increases both in urban and rural areas. Take the two-member group as an example, in which the nutrient intakes of urban and rural husbands increase by 0.055% and 0.048%, respectively, for every 1% increase in FDI openness. A 1% increase in FDI openness is followed by a 0.103% and 0.111% increase in the nutrient intake of the wife in urban and rural areas. The stronger labor time effect illustrates that physical activities resulting from longer labor time indeed require more nutrients to sustain them [20,32]. Evidence is provided to suggest that intrahousehold nutrient intakes in rural areas are more significant to FDI openness than urban areas. Both consumption patterns and physical activity levels are affected by lifestyles in rural areas that are relatively more conservative and traditional [32].

#### 4.4.3. FDI and Food Price Interaction Effect

Food price reduction enhances poor people’s access to more food [36] and has beneficial impacts on people’s nutritional well-being [42]. Bhargava [43] also documented that a high food price leads to an energy intake reduction. It is noteworthy that FDI can promote agriculture production [36] and thus influence food price. Despite the potential price effect brought by FDI on nutrition intake at the macro-level, the nutritional effect through the individual’s behavior based on the micro level is considered. Therefore, an interaction term between the accumulated food price and FDI is included to capture the possibility that the impact of a food price shock on the family role’s nutrient intake may vary with FDI. The accumulated food price index is constructed as in the above indices in Section 2 (e.g., *Pgrain*).

Table A3 shows the results of the food price interaction effect on calorie intake. The coefficients of the interaction term for the wife, son, and granddaughter are significantly negative in urban samples. When the price is rising, their calorie intake declines, responding to the FDI openness effect. The interaction terms in rural samples, however, show positive signs at the 1% level. This signifies that FDI and the price effect might jointly strengthen calorie intake.

#### 4.4.4. Intra-Household Intake Ratio Results

Nutrient intake ratio variation can determine the nutrient intake of the FDI effect for each role. Therefore, this study uses the intrahousehold calorie intake ratios (*Calorie ratio*) of each family role as independent variables instead.

The results of the intrahousehold nutrient intake ratio for each family role using the joint consumption analysis are listed in Table A4. Most coefficients of intrahousehold nutrient intake ratios are insignificant to the FDI effect due to little variation. However, the nutrient intake ratio of the son is significantly negative for every 1% increase in FDI in urban areas, while the coefficients are positive in rural areas. The evidence shows that the intrahousehold son bias may still be prevalent in Chinese rural families [62]. Ding and Zhang [62] illustrated that, due to Chinese social customs, sons should take care of their aged parents. In the family, the son is a positive shock on the expectation of the permanent income of the parents. The parents could regard their son as additional pension income.

#### 4.4.5. International Trade Effect Results

International trade openness is also a part of economic openness. Therefore, we include the international trade effect of nutrient intake in the joint consumption analysis. The *FDI* variable is replaced with the *Trade* variable in Table A5. The *Trade* variable is defined as the sum of imports and exports divided by gross domestic product for every province [36,63].

Compared to Table 4 and Table 5, the international trade openness also has a significant positive effect on the calorie intake of different family roles including husband, wife, son, and daughter. Actually, trade openness policies have stimulated the consumption patterns associated with nutrition transition and strengthened nutritional outcomes [7]. Trade theories also suggest that increasing international trade should benefit women, particularly those in developing countries because they are often considered unskilled labor [63]. Like the FDI effect, the international trade effect becomes insignificant as the number of family members increases. For the granddaughter in urban areas, international trade reduces calorie intake at a 10% level while the FDI effect is insignificant. Roemling and Qaim [32] noted that in the dual burden household, children are more likely to be underweight than adults. These results have reinforced what we discussed previously and made our findings more reliable.

## 5. Conclusions

This article utilizes CHNS data to analyze the impacts of economic openness (especially FDI) on intra-household nutrient intake. Several interesting and important conclusions were found, as follows.

(1) FDI openness has a positive and statistically significant impact on the labor force’s calorie intake. Policies on nutrition intake improvement should target more at the individual (according to family role within the household) level rather than the household level. Overall, the government should continue to encourage and attract FDI inflows since FDI plays a positive role in improving nutrient intake. However, with the increase in family size (e.g., in the five-member group), the impact of FDI on nutrient intake for some family roles showed less significance. This implies the existence of intrahousehold redistribution, and the level of effectiveness will decrease with family size.
(2) FDI openness and international trade openness both show positive signs on nutrient intake, especially in the rural areas. The rural intrahousehold nutrient intakes following the FDI openness effect are more significant even in the multi-numbers groups. Mussa [14] noted that intrahousehold inequalities are larger in rural areas than in urban areas. Our empirical evidence suggests that family members in rural areas can benefit more in terms of nutrient intake. This could help narrow the gap when attracting FDI inflows. Therefore, FDI in secondary industries, especially in the manufacturing industry, should be placed at a premium. For both rural labor employment and nutrition improvement, a certain proportion of FDI in the second industry should continue to be maintained by the government since more rural populations are employed in the manufacturing industry.
(3) FDI openness could change the non-income effect of nutrient intake on the urban labor force while affecting both income and non-income effects of nutrient intake on the rural labor force. As far as the non-income effect on nutrient intake is concerned, policies should target the promotion of nutrition education courses more widely, especially in rural areas. Those courses could spread health knowledge, encourage a healthy diet, and decrease the possibility of suffering from diet-related diseases.

## Figures and Tables

**Table 1:** Variables definition.

Variable	Definition
Income	
Hinc	Total household income (CNY in year 2011 price)
Inc.	Individual total net income (CNY in year 2011 price)
Nutrition	
Calorie	Three-day average calories intake (kcal)
Carbo (g)	Three-day average carbohydrate intake (g)
Protein (g)	Three-day average protein intake (g)
Fat (g)	Three-day average fat intake (g)
Calorie ratio	Intrahousehold calorie intake ratio (%)
Individual characteristics	
*Edu*	Average education years completed in a regular school (years)
*Age*	Average age (years)
*BMI*	Body mass index, weight/height^2^ (kg/m^2^)
Age dummies	
Preschool	(Age between 2 and 5 years old) = 1, others = 0
School	(Age between 6 and 17 years old) = 1, others = 0
Adult	(Age between 18 and 65 years old) = 1, others = 0
Old	(Age between 66 and 80 years old) = 1, others = 0
Highold	(Age above 81 years old) = 1, others = 0
Household characteristics	
Fsize	Number of people in a household (Heads)
Price shock	
Pgrain	Price index of grain based on year 2011
Poil	Price index of oil based on year 2011
Pmeat	Price index of meat and poultry based on year 2011
Pegg	Price index of egg based on year 2011
Paquatic	Price index of aquatic product based on year 2011
Pvegetable	Price index of vegetables based on year 2011
Openness degree	
FDI	Total FDI stock /GDP ratio at provincial level
IV for income	
Ktrans	Total asset value of transportation carriers (CNY in year 2011 price)
Kagr	Total asset value of agriculture appliances (CNY in year 2011 price)
Kprof	Total asset of professional appliances (CNY in year 2011 price)
Wage_male	Daily wage of male factory worker (CNY in year 2011 price)
Wage_female	Daily wage of female factory worker (CNY in year 2011 price)
Wage_helper	Daily wage of domestic helper (CNY in year 2011 price)
Wage_const	Daily wage of construction worker (CNY in year 2011 price)
Wage_driver	Monthly wage of driver (CNY in year 2011 price)

**Table 2:** Variables define and descriptive statistics.

Variable	Urban		Rural		Total	
	Mean	S.D.	Mean	S.D.	Mean	S.D.
Income						
Hinc (yuan)	32,945.51	36,529.98	22,730.82	31,397.60	26,129.45	33,540.39
Inc. (yuan)	9225.70	16,847.03	6039.68	14,322.04	7099.73	15,282.58
Time						
Time (hours)	1523.17	1457.87	1534.43	1503.04	1530.68	1488.17
Nutrition						
Calorie (kcal)	2105.00	1032.76	2230.62	872.09	2189.16	930.49
Carbo (g)	282.89	111.05	344.73	135.55	324.16	131.20
Protein (g)	76.01	94.13	63.67	62.72	66.37	25.78
Fat (g)	68.20	28.52	65.46	24.24	67.77	74.88
Calorie ratio	0.36	0.17	0.35	0.19	0.36	0.18
Individual characteristics						
Age (years)	42.10	19.95	39.28	19.96	40.21	20.00
Edu (years)	7.80	4.38	6.01	3.89	6.66	4.13
BMI (kg/m^2^)	22.24	3.94	21.53	3.98	21.76	3.98
Age dummies						
Preschool	0.01	0.07	0.01	0.09	0.01	0.09
School	0.14	0.35	0.18	0.38	0.17	0.37
Adult	0.72	0.45	0.70	0.46	0.71	0.46
Old	0.12	0.32	0.09	0.29	0.10	0.30
Highold	0.02	0.14	0.01	0.12	0.02	0.12
Household characteristics						
*Fsize* (heads)	3.68	1.39	4.17	1.58	4.01	1.54
Price shock						
Pgrain	0.60	0.26	0.57	0.26	0.58	0.26
Poil	0.68	0.21	0.65	0.21	0.66	0.21
Pmeat	0.58	0.25	0.54	0.24	0.55	0.25
Pegg	0.61	0.25	0.58	0.24	0.59	0.24
Paquatic	0.70	0.19	0.67	0.19	0.68	0.19
Pvegetable	0.55	0.29	0.51	0.28	0.52	0.28
Openness degree						
FDI (ratio)	0.07	0.06	0.06	0.05	0.07	0.06
IV for income						
Ktrans (yuan)	8992.64	47,025.33	6689.39	36,114.24	7455.72	40,090.15
Kagr (yuan)	228.99	2098.29	1127.49	5492.16	828.54	4666.02
Kprof (yuan)	255.75	3828.78	486.82	5216.03	409.94	4800.40
Wage_male	32.52	64.55	26.09	25.98	28.23	42.97
Wage_female	27.72	61.63	21.48	31.44	23.56	43.95
Wage_helper	21.27	61.43	11.75	27.03	14.91	41.99
Wage_const	40.58	50.37	36.59	37.81	37.92	42.45
Wage_driver	39.60	39.68	34.34	33.18	36.09	35.56

**Table 3:** Family role regressions results (dependent variable: ln individual calorie).

**Urban**	**Husband**	**Son**	**Father**	**Son-In-Law**	**Grandson**
*lnFDI*	0.084 ***	0.003	−0.028	0.107	−0.014
R^2^	0.131	0.142	0.429	0.488	0.378
N	9531	4634	181	142	641
	**Wife**	**Daughter**	**Mather**	**Dau-In-Law**	**Granddaughter**
*lnFDI*	0.107 ***	−0.022	0.091	0.147 *	−0.052
R^2^	0.119	0.120	0.169	0.131	0.214
N	10,471	3367	490	1208	626
**Rual**	**Husband**	**Son**	**Father**	**Son-In-Law**	**Grandson**
*lnFDI*	0.096 ***	0.084 ***	0.106	0.133	−0.047
R^2^	0.122	0.183	0.213	0.325	0.318
N	18,513	10,551	433	143	1244
	**Wife**	**Daughter**	**Mather**	**Dau-In-Law**	**Granddaughter**
*lnFDI*	0.113 ***	0.113 ***	0.011	0.111 **	−0.034
R^2^	0.147	0.112	0.216	0.108	0.238
N	20,300	6972	1088	2618	1070

Notes: * Significant at the 10% level; ** significant at the 5% level; *** significant at the 1% level. Dau-in-law refers to Daughter-in-law.

**Table 4:** Urban multiple members’ joint consumption effect (dependent variable: ln individual calorie, carbohydrate, fat and protein).

Family Role	2-Members	3-Members		4-Members		5-Members	
*Calorie*							
Husband	0.058 ***	0.042	0.038	0.024	0.059	0.030	0.017
R^2^	0.184	0.205	0.231	0.232	0.209	0.420	0.368
Wife	0.051 ***	0.067 **	0.030	0.080	0.120	0.200	−0.163
R^2^	0.167	0.213	0.220	0.226	0.229	0.461	0.363
Son	-	−0.016	-	−0.045	−0.022	0.024	−0.173
R^2^	-	0.281	-	0.399	0.312	0.454	0.534
Daughter	-	-	−0.045	−0.015	-	-	-
R^2^	-	-	0.198	0.229	-	-	-
Dau-in-law	-	-	-	-	0.063	0.056	−0.020
R^2^	-	-	-		0.276	0.455	0.449
Grandson	-	-	-	-	-	0.087	-
R^2^	-	-	-	-	-	0.790	-
Granddaughter	-	-	-	-	-	-	−0.042
R^2^	-	-	-	-	-	-	0.398
*Carbohydrate*							
Husband	0.059 ***	0.062 *	0.038	0.074	0.119	0.087	0.070
R^2^	0.282	0.318	0.360	0.336	0.300	0.405	0.445
Wife	0.064 ***	0.083 ***	0.045	0.110 *	0.138	0.423 *	−0.140
R^2^	0.289	0.346	0.389	0.390	0.328	0.482	0.445
Son	-	−0.002	-	0.016	−0.009	0.022	−0.156
R^2^	-	0.331	-	0.446	0.360	0.431	0.544
Daughter	-	-	−0.014	0.039	-	-	-
R^2^	-	-	0.320	0.323	-	-	-
Dau-in-law	-	-	-	-	0.114	0.216	0.041
R^2^	-	-	-	-	0.382	0.478	0.519
Grandson	-	-	-	-	-	0.097	-
R^2^	-	-	-	-	-	0.547	-
Granddaughter	-	-	-	-	-	-	0.006
R^2^	-	-	-	-	-	-	0.383
*Fat*							
Husband	−0.038	−0.063	−0.057	−0.073	−0.136	−0.262	−0.411
R^2^	0.106	0.136	0.129	0.197	0.139	0.462	0.453
Wife	−0.017	0.009	−0.079	−0.035	0.043	−0.337	−0.514
R^2^	0.102	0.152	0.140	0.208	0.176	0.468	0.426
Son	-	−0.111 **	-	−0.246 **	−0.134	−0.218	−0.581 *
R^2^	-	0.208	-	0.294	0.187	0.451	0.484
Daughter	-	-	−0.159 **	−0.180	-	-	-
R^2^	-	-	0.175	0.260	-	-	-
Dau-in-law	-	-	-	-	−0.006	−0.206	−0.383
R^2^	-	-	-	-	0.164	0.382	0.435
Grandson	-	-	-	-	-	0.106	-
R^2^	-	-	-	-	-	0.517	-
Granddaughter	-	-	-	-	-	-	−0.463
R^2^	-	-	-	-	-	-	0.565
*Protein*							
Husband	0.092 ***	0.060 *	0.092 ***	0.051	0.149 *	0.059	0.236
R^2^	0.129	0.137	0.125	0.131	0.152	0.381	0.363
Wife	0.071 ***	0.066 **	0.053	0.058	0.168 *	0.342 *	0.196
R^2^	0.105	0.132	0.101	0.121	0.164	0.459	0.338
Son	-	−0.014	-	−0.089	0.061	0.164	0.189
R^2^	-	0.247	-	0.351	0.205	0.452	0.445
Daughter	-	-	−0.028	−0.076	−	−	−
R^2^	−	−	0.162	0.197	−	−	−
Dau-in-law	−	−	−	−	0.129	0.110	0.262
R^2^	−	−	−	−	0.169	0.435	0.342
Grandson	−	−	−	−	−	0.217	−
R^2^	−	−	−	−	−	0.605	−
Granddaughter	−	−	−	−	−	−	0.153
R^2^	−	−	−	−	−	−	0.525
N	8841	3319	2485	771	657	160	156

Notes: * Significant at the 10% level; ** significant at the 5% level; *** significant at the 1% level. Dau-in-law refers to Daughter-in-law.

**Table 5:** Rural multiple members’ joint consumption effect (dependent variable: ln individual calorie, carbohydrate, fat and protein).

Family Role	2-Members	3-Members		4-Members		5-Members	
*Calorie*							
Husband	0.102 ***	0.080 ***	0.048 **	0.002	0.164 ***	0.279 **	0.226 *
R^2^	0.175	0.180	0.189	0.191	0.191	0.362	0.344
Wife	0.107 ***	0.074 ***	0.081 ***	0.041	0.147 ***	0.209 *	0.193
R^2^	0.196	0.201	0.226	0.207	0.186	0.303	0.304
Son	-	0.105 ***	-	0.077 **	0.134 **	0.221 *	0.170
R^2^	-	0.321	-	0.395	0.147	0.255	0.263
Daughter	-	-	0.100 ***	0.045	-	-	-
R^2^	-	-	0.327	0.327	-	-	-
Dau-in-law	-	-	-	-	0.153 ***	0.205	−0.037
R^2^	-	-	-	-	0.200	0.261	0.329
Grandson	-	-	-	-	-	−0.039	-
R^2^	-	-	-	-	-	0.432	-
Granddaughter	-	-	-	-	-	-	−0.052
R^2^	-	-	-	-	-	-	0.432
*Carbohydrate*							
Husband	0.075 ***	0.054 **	0.041	0.003	0.193 ***	0.302 **	0.190
R^2^	0.291	0.293	0.324	0.290	0.330	0.494	0.480
Wife	0.093 ***	0.059 ***	0.068 ***	0.032	0.199 ***	0.242 *	0.248 *
R^2^	0.312	0.320	0.366	0.310	0.315	0.457	0.422
Son	-	0.068 ***	-	0.065 *	0.148 **	0.130	0.121
R^2^	-	0.355	-	0.431	0.321	0.398	0.455
Daughter	-	-	0.078 ***	0.023	-	-	-
R^2^	-	-	0.409	0.377	-	-	-
Dau-in-law	-	-	-	-	0.206 ***	0.211	−0.007
R^2^	-	-	-	-	0.376	0.439	0.531
Grandson	-	-	-	-	-	−0.044	-
R^2^	-	-	-	-	-	0.461	-
Granddaughter	-	-	-	-	-	-	−0.132
R^2^	-	-	-	-	-	-	0.408
							
*Fat*							
Husband	0.076 ***	0.071 *	0.014	−0.002	−0.005	0.241	0.211
R^2^	0.120	0.133	0.121	0.108	0.193	0.258	0.318
Wife	0.109 ***	0.098 **	0.090 *	0.066	−0.011	0.062	0.029
R^2^	0.130	0.145	0.131	0.128	0.229	0.293	0.309
Son	-	0.158 ***	-	0.116	0.025	0.202	0.087
R^2^	-	0.207	-	0.182	0.197	0.294	0.250
Daughter	-	-	0.123 **	0.084	-	-	-
R^2^	-	-	0.176	0.172	-	-	-
Dau-in-law	-	-	-	-	−0.015	0.008	−0.153
R^2^	-	-	-	-	0.184	0.243	0.252
Grandson	-	-	-	-	-	−0.080	-
R^2^	-	-	-	-	-	0.433	-
Granddaughter	-	-	-	-	-	-	0.135
R^2^	-	-	-	-	-	-	0.414
*Protein*							
Husband	0.131 ***	0.105 ***	0.094 ***	0.002	0.271 ***	0.311 **	0.408 ***
R^2^	0.158	0.138	0.144	0.166	0.182	0.315	0.295
Wife	0.137 ***	0.097 ***	0.113 ***	0.028	0.243 ***	0.273 *	0.430 ***
R^2^	0.156	0.145	0.158	0.168	0.168	0.254	0.288
Son	-	0.132 ***	-	0.074 *	0.211 ***	0.253 *	0.403 ***
R^2^	-	0.295	-	0.355	0.124	0.217	0.219
Daughter	-	-	0.132 ***	0.045	-	-	-
R^2^	-	-	0.280	0.294	-	-	-
Dau-in-law	-	-	-	-	0.233 ***	0.250 *	0.198
R^2^	-	-	-	-	0.146	0.215	0.237
Grandson	-	-	-	-	-	−0.003	-
R^2^	-	-	-	-	-	0.418	-
Granddaughter	-	-	-	-	-	-	0.068
R^2^	-	-	-	-	-	-	0.401
N	16,774	6977	4661	2419	1318	326	284

Notes: * Significant at the 10% level; ** significant at the 5% level; *** significant at the 1% level. Dau-in-law refers to daughter-in-law.

**Table 6:** Urban multiple adult members’ individual income effect.

	**2-Members**		**3-Members I**			**3-Members II**		
	**Husband**	**Wife**	**Husband**	**Wife**	**Son**	**Husband**	**Wife**	**Daughter**
*The 1st step (dependent variable: ln individual income)*								
*lnFDI*	−0.155	−0.365	−1.028	0.815	−0.852	−1.364	−1.696	1.030
*The 2nd step (dependent variable: ln calorie intake)*								
*lnFDI*	0.077 ***	0.058 ***	0.059	0.044	0.010	0.050	0.041	−0.007
*Hus_inc*	0.007 ***	0.006 ***	0.008 ***	0.003	0.001	0.008 **	0.008 *	0.008 *
*Wife_inc*	0.003 *	0.001	0.006 *	0.006 **	0.009 ***	0.000	−0.001	−0.006 *
*Son_inc*	-	-	0.001	0.002	0.003	-	-	-
*Dau_inc*	-	-	-	-	-	0.000	−0.004	−0.003
R^2^	0.168	0.169	0.167	0.170	0.175	0.189	0.193	0.197
N	6250	6250	1139	1139	1139	797	797	797
	**4-members I**				**4-members II**			
	**Husband**	**Wife**	**Son**	**Daughter**	**Husband**	**Wife**	**Son**	**D.-in-law**
*The 1st step (dependent variable: ln individual income)*								
*lnFDI*	2.671	4.550	0.384	−3.205	−1.134	−0.602	−3.911	3.589
*The 2nd step (dependent variable: ln calorie intake)*								
*lnFDI*	0.004	0.177	−0.002	0.117	−0.025	0.028	−0.066	−0.043
*Hus_inc*	0.003	−0.000	−0.002	0.002	−0.002	−0.003	−0.004	−0.006
*Wife_inc*	0.007	0.009	0.012	0.009	0.002	0.002	0.008	0.005
*Son_inc*	−0.007	−0.017 **	−0.005	−0.006	0.005	0.004	0.007	−0.003
*Dau_inc*	−0.001	−0.000	0.001	−0.005	-	-	-	-
*DIL_inc*	-	-	-	-	−0.002	0.002	−0.004	−0.003
R^2^	0.234	0.278	0.305	0.255	0.214	0.285	0.337	0.377
N	177	177	177	177	274	274	274	274

Note: * Significant at the 10% level; ** significant at the 5% level; *** significant at the 1% level. Hus refers to husband, DIL and D.-in-law both refer to daughter-in-law.

**Table 7:** Rural multiple adult members’ individual income effect.

**Family Role**	**2-Members**		**3-Members I**			**3-Members II**		
	**Husband**	**Wife**	**Husband**	**Wife**	**Son**	**Husband**	**Wife**	**Daughter**
*The 1st step (dependent variable: ln individual income)*								
*lnFDI*	0.469 ***	0.310 **	0.915 *	0.658	0.971	−0.031	0.493	−0.020
*The 2nd step (dependent variable: ln calorie intake)*								
*lnFDI*	0.089 ***	0.106 ***	0.068 *	0.079 **	0.067 *	0.050	0.134 **	0.118 **
*Hus_inc*	0.001	−0.002	0.002	−0.006 **	0.000	−0.001	0.000	−0.003
*Wife_inc*	0.007 ***	0.009 ***	0.006 **	0.009 ***	0.003	0.003	0.006 **	0.002
*Son_inc*	-	-	0.005 **	0.003	0.004 **	-	-	-
*Dau_inc*	-	-	-	-	-	−0.000	−0.001	−0.002
R^2^	0.156	0.201	0.181	0.211	0.188	0.212	0.261	0.310
N	12,886	12,886	2196	2196	2196	1240	1240	1240
	**4−members I**				**4−members II**			
	**Husband**	**Wife**	**Son**	**Daughter**	**Husband**	**Wife**	**Son**	**D.-in-law**
*The 1st step (dependent variable: ln individual income)*								
*lnFDI*	−0.303	4.083 **	2.167	0.749	−1.347	2.806 *	1.525	−2.873
*The 2nd step (dependent variable: ln calorie intake)*								
*lnFDI*	0.014	0.105	0.099	0.099	0.055	0.074	0.077	0.181 **
*Hus_inc*	0.002	0.006	0.001	0.006	−0.006	−0.012 ***	−0.013 ***	−0.016 ***
*Wife_inc*	−0.004	−0.001	−0.009 *	−0.013 **	0.006	0.006	0.003	0.004
*Son_inc*	−0.001	−0.001	−0.000	−0.004	0.005	0.003	0.009 *	0.014 *
*Dau_inc*	0.000	0.001	−0.002	−0.004	-	-	-	-
*DIL_inc*	-	-	-	-	−0.003	−0.001	−0.005	−0.008 *
R^2^	0.318	0.301	0.377	0.422	0.191	0.211	0.203	0.264
N	397	397	397	397	687	687	687	687

Notes: * Significant at the 10% level; ** significant at the 5% level; *** significant at the 1% level. Hus refers to husband, DIL and D.-in-law both refer to daughter-in-law.

## Appendix A

**Table A1:** Regression results of urban roles with age dummies (dependent variable: ln individual calorie).

**Urban**	**Husband**	**Son**	**Father**	**Son-In-Law**	**Grandson**
*lnFDI*	0.083 ***	0.002	−0.039	0.043	−0.001
R^2^	0.131	0.115	0.439	0.485	0.376
N	9531	4634	181	142	641
	**Wife**	**Daughter**	**Mother**	**Dau-In-Law**	**Granddaughter**
*lnFDI*	0.106 ***	−0.036	0.093	0.154 *	−0.060
R^2^	0.120	0.105	0.169	0.129	0.192
N	10471	3367	490	1208	626
**Rural**	**Husband**	**Son**	**Father**	**Son-In-Law**	**Grandson**
*lnFDI*	0.102 ***	0.100 ***	0.096	0.307	−0.038
R^2^	0.123	0.152	0.213	0.283	0.315
N	18513	10551	433	143	1244
	**Wife**	**Daughter**	**Mother**	**Dau-In-Law**	**Granddaughter**
*lnFDI*	0.119 ***	0.110 ***	0.010	0.106 **	−0.013
R^2^	0.148	0.091	0.218	0.109	0.244
N	20300	6972	1088	2618	1070

Notes: * Significant at the 10% level; ** significant at the 5% level; *** significant at the 1% level. Dau-in-law refers to daughter-in-law.

**Table A2:** Labor time-controlled effect regression results (dependent variable: Ln individual calorie).

Family Role	2-Members	3-Members		4-Members	
**Urban**					
Husband	0.055 ***	0.026	0.034	0.097	−0.013
R^2^	0.179	0.206	0.230	0.281	0.237
Wife	0.048 **	0.049	0.059	0.304 ***	0.100
R^2^	0.170	0.203	0.219	0.256	0.251
Son	-	−0.004	-	0.079	−0.052
R^2^	-	0.217	-	0.297	0.323
Daughter	-	-	−0.017	0.099	-
R^2^	-	-	0.186	0.223	-
Dau-in-law	-	-	-	-	0.064
R^2^	-	-	-	-	0.292
N	7530	1521	1196	272	478
**Rural**					
Husband	0.103 ***	0.096 ***	0.075 **	−0.041	0.220 ***
R^2^	0.171	0.195	0.226	0.262	0.201
Wife	0.111 ***	0.106 ***	0.145 ***	0.041	0.168 ***
R^2^	0.200	0.211	0.266	0.291	0.208
Son	-	0.086 ***	-	0.040	0.176 ***
R^2^	-	0.217	-	0.323	0.176
Daughter	-	-	0.156 ***	0.027	-
R^2^	-	-	0.343	0.359	-
Dau-in-law	-	-	-	-	0.182 ***
R^2^	-	-	-	-	0.225
N	15,320	3190	2159	686	1036

Notes: * Significant at the 10% level; ** significant at the 5% level; *** significant at the 1% level. Dau-in-law refers to daughter-in-law.

**Table A3:** FDI and food price interaction term results (dependent variable: ln individual calorie).

Family Role	2-Members	3-Members		4-Members		5-Members	
**Urban**							
Husband	0.032	0.037	0.005	0.062	0.061	0.227	−0.174
R^2^	0.181	0.203	0.223	0.220	0.200	0.404	0.334
Wife	−0.022	0.013	−0.078 **	0.029	−0.052	0.145	−0.292
R^2^	0.165	0.209	0.214	0.220	0.221	0.436	0.359
Son	-	−0.056	-	0.041	0.017	0.217	−0.493 ***
R^2^	-	0.280	-	0.394	0.299	0.410	0.517
Daughter	-	-	−0.042	0.042	-	-	-
R^2^	-	-	0.192	0.223	-	-	-
Dau-in-law	-	-	-	-	0.045	0.360 **	−0.277
R^2^	-	-	-	-	0.263	0.405	0.430
Grandson	-	-	-	-	-	0.520 ***	−
R^2^	-	-	-	-	-	0.595	−
Granddaughter	-	-	-	-	-	-	−0.405 **
R^2^	-	-	-	-	-	-	0.375
N	8841	3319	2485	771	657	160	156
**Rural**							
Husband	0.135 ***	0.172 ***	0.13***	0.140 ***	0.149**	0.155	0.198
R^2^	0.174	0.180	0.185	0.183	0.187	0.343	0.336
Wife	0.083 ***	0.105 ***	0.114 ***	0.159 ***	0.077	0.042	0.196
R^2^	0.194	0.198	0.223	0.202	0.176	0.267	0.291
Son	-	0.152 ***	−	0.165 ***	0.163 ***	0.180	0.350 ***
R^2^	-	0.319	−	0.391	0.137	0.234	0.254
Daughter	-	-	0.094 ***	0.125 ***	-	-	-
R^2^	-	-	0.323	0.325	-	-	-
Dau-in-law	-	-	-	-	0.100	0.093	0.211
R^2^	-	-	-	-	0.187	0.233	0.302
Grandson	-	-	-	-	-	−0.139	-
R^2^	-	-	-	-	-	0.423	-
Granddaughter	-	-	-	-	-	-	0.054
R^2^	-	-	-	-	-	-	0.429
N	16,774	6977	4661	2419	1318	326	284

Notes: * Significant at 10%; ** significant at 5% level; *** significant at 1% level. Dau-in-law refers to daughter-in-law.

**Table A4:** Intra-household intake ratio regression results (dependent variable: ln individual calorie ratio).

Family Role	2-Members	3-Members		4-Members		5-Members	
**Urban**							
Husband	0.006	−0.014	0.022	−0.000	−0.069	−0.076	−0.006
R^2^	0.642	0.601	0.563	0.530	0.410	0.499	0.462
Wife	0.014	0.023 *	0.030 *	0.051 *	0.006	0.094	−0.183
R^2^	0.636	0.608	0.552	0.572	0.408	0.497	0.523
Son	-	−0.078 ***	-	−0.071 *	−0.132 ***	−0.082	−0.192 *
R^2^	-	0.545	-	0.511	0.333	0.474	0.585
Daughter	-	-	−0.045 **	−0.041	-	-	-
R^2^	-	-	0.507	0.439	-	-	-
Dau-in-law	-	-	-	-	−0.036	−0.050	−0.044
R^2^	-	-	-	-	0.330	0.471	0.469
Grandson	-	-	-	-	-	−0.019	-
R^2^	-	-	-	-	-	0.715	-
Granddaughter	-	-	-	-	-	-	−0.065
R^2^	-	-	-	-	-	-	0.632
N	8841	3319	2485	771	657	160	156
**Rural**							
Husband	−0.001	0.014	−0.039 ***	−0.039 **	0.067 **	0.097	0.078
R^2^	0.574	0.594	0.519	0.514	0.452	0.465	0.505
Wife	0.006	0.010	−0.002	0.002	0.039	0.028	0.045
R^2^	0.573	0.599	0.536	0.552	0.441	0.458	0.435
Son	-	0.040 ***	-	0.037 *	0.027	0.042	0.024
R^2^	-	0.518	-	0.525	0.392	0.492	0.439
Daughter	-	-	0.011	0.005	-	-	-
R^2^	-	-	0.472	0.443	-	-	-
Dau-in-law	-	-	-	-	0.009	0.025	−0.186 **
R^2^	-	-	-	-	0.342	0.478	0.440
Grandson	-	-	-	-	-	−0.227	-
R^2^	-	-	-	-	-	0.616	-
Granddaughter	-	-	-	-	-	-	−0.196 *
R^2^	-	-	-	-	-	-	0.627
N	16,774	6977	4661	2419	1318	326	284

Notes: * Significant at the 10% level; ** significant at the 5% level; *** significant at the 1% level. Dau-in-law refers to daughter-in-law.

**Table A5:** International trade effect results (dependent variable: ln individual calorie).

Family Role	2-Members	3-Members		4-Members		5-Members	
**Urban**							
Husband	0.022 ***	0.024 *	0.012	−0.013	0.029	−0.037	−0.054
R^2^	0.183	0.206	0.230	0.232	0.209	0.421	0.370
Wife	−0.003	0.007	−0.007	−0.013	−0.012	−0.047	−0.103
R^2^	0.167	0.211	0.220	0.224	0.226	0.456	0.365
Son	-	0.000	-	−0.016	0.012	−0.086	−0.054
R^2^	-	0.281	-	0.399	0.312	0.461	0.531
Daughter	-	-	−0.017	−0.010	-	-	-
R^2^	-	-	0.198	0.229	-	-	-
Dau-in-law	-	-	-	-	−0.014	−0.095	−0.087
R^2^	-	-	-	-	0.276	0.462	0.453
Grandson	-	-	-	-	-	−0.069	-
R^2^	-	-	-	-	-	0.591	-
Granddaughter	-	-	-	-	-	-	−0.190 *
R^2^	-	-	-	-	-	-	0.418
N	8841	3319	2485	771	657	160	156
**Rural**							
Husband	0.067 ***	0.046 ***	0.044 ***	0.015	0.067 ***	0.183 **	0.061
R^2^	0.177	0.181	0.190	0.191	0.190	0.364	0.338
Wife	0.049 ***	0.024 **	0.035 ***	0.012	0.036	0.115	−0.003
R^2^	0.196	0.200	0.225	0.207	0.182	0.302	0.297
Son	-	0.043 ***	-	0.012	0.030	0.141 *	0.038
R^2^	-	0.320	-	0.394	0.144	0.256	0.259
Daughter	-	-	0.038 ***	0.013	-	-	-
R^2^	-	-	0.326	0.326	-	-	-
Dau-in-law	-	-	-	-	0.030	0.097	−0.014
R^2^	-	-	-	-	0.196	0.258	0.329
Grandson	-	-	-	-	-	0.031	-
R^2^	-	-	-	-	-	0.432	-
Granddaughter	-	-	-	-	-	-	−0.046
R^2^	-	-	-	-	-	-	0.433
N	16,774	6977	4661	2419	1318	326	284

Notes: * Significant at 10%; ** significant at 5% level; *** significant at 1% level. Dau-in-law refers to daughter-in-law.
